# The influence of depth of sedation on motor evoked potentials monitoring in youth from 4 to 23 years old: preliminary data from a prospective observational study

**DOI:** 10.3389/fmed.2024.1471450

**Published:** 2024-10-29

**Authors:** Jan Hudec, Martina Kosinová, Tereza Prokopová, Hana Zelinková, Kamil Hudáček, Martin Repko, Roman Gál, Petr Štourač

**Affiliations:** ^1^Department of Anesthesiology and Intensive Care Medicine, University Hospital Brno and Faculty of Medicine, Masaryk University, Brno, Czechia; ^2^Department of Simulation Medicine, Faculty of Medicine, Masaryk University, Brno, Czechia; ^3^Department of Pediatric Anesthesiology and Intensive Care Medicine, University Hospital Brno and Faculty of Medicine, Masaryk University, Brno, Czechia; ^4^Institute of Biostatistics and Analyses, Faculty of Medicine, Brno, Czechia; ^5^Department of Orthopedic Surgery, University Hospital Brno and Faculty of Medicine, Masaryk University, Brno, Czechia

**Keywords:** bispectral index, intraoperative neurophysiological monitoring, motor evoked potentials, remifentanil, scoliosis surgery, total intravenous anesthesia

## Abstract

**Introduction:**

The influence of various levels of sedation depth on motor evoked potentials (MEP) reproducibility in youth is still unclear because of a lack of data. We tested the hypothesis that a deeper level of total intravenous anesthesia (TIVA) [bispectral index (BIS) 40 ± 5 compared to 60 ± 5] can affect surgeon-directed MEP and their interpretation in youths.

**Methods:**

All patients received TIVA combined with propofol and remifentanil. TIVA was initially maintained at a BIS level of 60 ± 5. The sedation anesthesia was deepened to BIS level 40 ± 5 before the skin incision. MEP were recorded and interpreted at both BIS levels. The primary endpoint was to evaluate the effect of the depth of sedation on the MEP reproducibility directed and interpreted by the surgical team in each patient separately. The secondary endpoint was to compare the relativized MEP parameters (amplitude and latency) in percentage at various levels of sedation in each patient separately. We planned to enroll 150 patients. Due to the COVID-19 pandemic, we decided to analyze the results of the first 50 patients.

**Results:**

The surgical team successfully recorded and interpreted MEP in all 50 enrolled patients in both levels of sedation depth without any clinical doubts. The MEP parameters at BIS level 40 ± 5, proportionally compared with the baseline, were latency 104% (97–110%) and the MEP amplitudes 84.5% (51–109%).

**Conclusion:**

Preliminary data predict that deeper sedation (BIS 40 ± 5) does not affect the surgical team’s interpretation of MEP in youth patients. These results support that surgeon-directed MEP may be an alternative when neurophysiologists are unavailable.

## 1 Introduction

The intraoperative neurophysiological monitoring (IONM) technique has become a standard in many surgical fields. This commonly used method enables high-risk surgeries by decreasing the potential risk of neurological injury. IONM includes motor evoked potentials (MEP), somatosensory evoked potentials (SSEP), brainstem auditory evoked potentials (BAEP), electromyography (EMG), and electroencephalography (EEG) (1–3).

The application of IONM has increased in the pediatric population, e.g., in scoliosis surgery, which is challenging for the perioperative team, especially for the risk of neurological injury (4–8). IONM is indicated to decrease the risk of new neurological deficit development. The Scoliosis Research Society strongly recommends its use during spinal deformity surgery (9). MEP and SSEP are IONM modalities commonly used in pediatric scoliosis surgery (10), and their monitoring replaced the intraoperative clinical examination, the wake-up test (11). The great advantage of IONM is that it detects possible nerve injuries during the instrumentation in time, which could more efficiently prevent them. Additionally, the wake-up test can be performed on a limited basis in pediatric patients. There is generally more difficulty in cooperation with pediatric patients, especially with developmental delays or neuromuscular disorders (10, 12).

Multimodal monitoring (SSEP and MEP) directed by neurophysiologists is an optimal way to detect neurological injury during surgeries (9, 13). It decreases the incidence of false-negative reports. Furthermore, the application of SSEP with MEP allows some level of neurophysiological monitoring even in cases when one of the mentioned modalities is not suitable or suspended for different reasons (e.g., MEP may not be suitable for patients with severe motor deficit; in contrast, SSEP could provide at least information about sensory pathways) (14, 15). On the other hand, surgeon-directed transcranial MEP monitoring represents a possible and safe alternative in cases when neurophysiologists are unavailable. However, the final decision for a particular type of monitoring should be made individually for each patient (14, 16).

During anesthesia, anesthetic agents and several pathophysiological conditions (severe hypoxia, hypercapnia, or hypotension) can significantly affect the IONM (17). The anesthetic team should ensure the conditions for optimal MEP monitoring. Total intravenous anesthesia (TIVA) with a combination of propofol and opioids represents the “gold standard” for IONM. The influence of anesthetic or analgesic agents on MEP was studied in several studies (18–20). However, the overall influence of the depth of TIVA sedation maintained at recommended levels is still unclear, especially in pediatric patients (2, 17).

This study aims to evaluate and describe the influence of the sedation depth guided by bispectral index (BIS) and targeted to recommended levels (BIS 40 ± 5 versus 60 ± 5) on MEP interpretation by surgeons and measured parameters (amplitude and latency) in youth. We aimed to assess the hypothesis that a deeper level of TIVA can affect surgeon-directed MEP and their interpretations by a surgical team. This hypothesis includes prolonged MEP latencies and decreased MEP amplitudes on a deeper level of sedation, BIS 40 ± 5.

## 2 Materials and methods

### 2.1 Study design

The Scoliosis (SCOL) study was designed as a prospective, monocentric before-after-trial. It was approved by the Ethics Committee of the University Hospital Brno, Czech Republic, on 24th June 2020 (No. 90/20, chair PharmD. Sarka Kozakova). We registered the study on the CilinicalTrials.gov registry (NCT04423146) on 5th June 2020. The project was managed according to international regulations and guidelines (Helsinki Declaration). We used the STROBE checklist to publish this article (21). According to the power analysis, the planned sample size was 150 patients. Power analysis was created with the following settings. We evaluated the width of the 95% confidence interval (CI) for the endpoint occurrence (non-reproducibility of MEP). The expected occurrence of the endpoint was 10%. However, due to the prolonged recruitment during the COVID-19 pandemic, we decided to perform a post-hoc power analysis and publish the results of the first 50 patients. The sample size of 50 produces a two-sided 95% confidence interval with a width equal to 0.185 when the sample proportion is 0.100.

### 2.2 Participants

The inclusion criteria were all children and youth diagnosed with congenital, neuromuscular, infantile idiopathic, juvenile idiopathic, adolescent idiopathic, or other syndromic scoliosis scheduled for elective scoliosis surgery in TIVA with surgeon-directed MEP monitoring. We determined childhood and teenage age according to orthopedic diagnostic criteria for not adult scoliosis surgery, according to skeletal maturity, specifically the Risser sign, because bone age better indicates biological maturity than chronological age (22). All participants or legal representatives could withdraw from the SCOL study at any time without giving any reason. Exclusion criteria were scoliosis surgery without surgeon-directed MEP monitoring, known contraindications for propofol or remifentanil administration (e.g., soy, egg lecithin, or peanut allergy), and inability to attach BIS or MEP electrodes to the standard positions. We excluded patients with known motor disease with no possibility of provoking MEP. All enrolled patients were managed according to the anesthesia and MEP monitoring local protocols (see below). The influence of depth of sedation on MEP was evaluated for each patient separately.

### 2.3 Anesthesia protocol

Before surgery, all enrolled patients were evaluated and premedicated with 0.1–0.2 mg/kg of midazolam *per os* (PO), eventually in combination with promethazine PO or atropine PO administered about 60 min before surgery. An intravenous (IV) bolus of propofol in dose 1.5–3 mg/kg, remifentanil in dose 1–2 μg/kg, and 0.6 mg/kg of rocuronium (or other intermediate-acting neuromuscular agents in appropriate dosing in case of rocuronium outage) were administered for the induction to anesthesia. Sevoflurane was administered for induction to anesthesia in case of a patient’s limited cooperation and the impossibility of IV line cannulation. Muscle relaxants could only be administered to facilitate endotracheal intubation. The anesthesia maintenance was performed with TIVA: propofol and remifentanil. The initial remifentanil infusion rate was about 0.1–0.2 μg/kg/min, and the initial propofol infusion rate started at about 4–5 mg/kg/h with titrating to BIS level 60 ± 5. The second IV line, arterial line, and permanent urine catheter were inserted after endotracheal intubation. In case of peripheral venous system insufficiency, the central venous catheter was administered. Also, surgeons applied electrodes for MEP monitoring after the anesthesia induction and airway securement. We used a BIS with standard frontotemporal EEG electrodes for depth of sedation anesthesia monitoring (BIS™ complete 2-channel Monitor, Covidien/Medtronic GmbH, Germany). The adequate level of sedation for surgery was targeted with BIS in combination with monitoring signs of light sedation (patients’ movements, increased heart rate and blood pressure or interference with artificial ventilation). The quantitative measurement of neuromuscular blockade, particularly Train-of-four ratio, was used to determine residual blockade (23). All the above was applied before turning the patient to the prone position. The remifentanil infusion rate was raised to 0.5–0.6 μg/kg/min, and the propofol infusion rate was raised to 5–7 mg/kg/h with titrating to BIS level 40 ± 5 before skin incision. The dosages of propofol and remifentanil were chosen according to the recommendations and pharmacokinetic studies (24–26). We titrated the TIVA between the BIS levels 40–60 during the surgery. The depth of the sedation and blood loss influence patients’ cardiovascular stability. Children’s vital functions, such as saturation, end-tidal CO_2_, heart rate, invasive blood pressure, and body temperature, were monitored and managed (e.g., administering vasopressors, fluid therapy, blood products, coagulation factors…) to maintain the values recommended in European Pediatric Advanced Life Support (EPALS) guidelines and EBM (27, 28).

### 2.4 Motor evoked potentials monitoring

**Figure 1:**
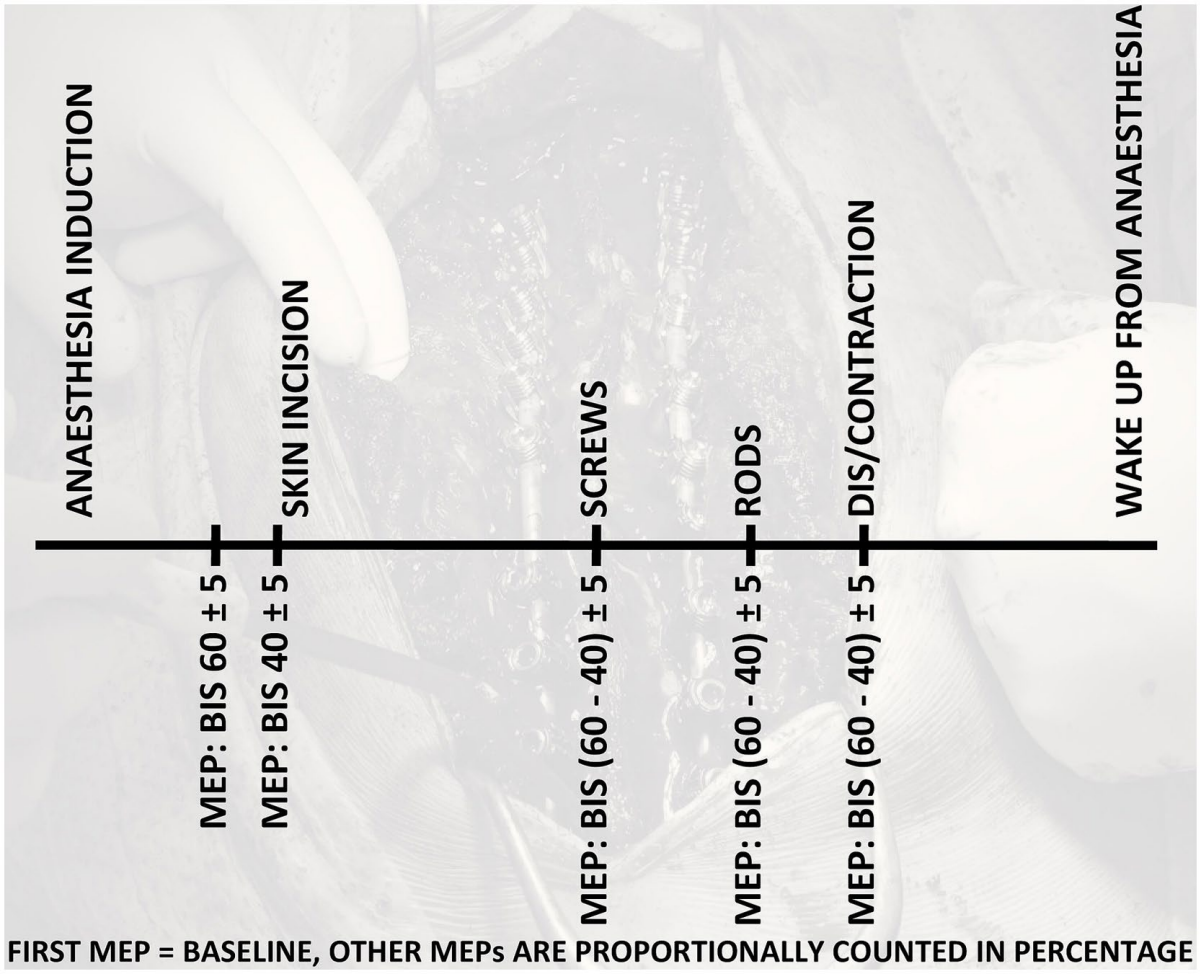
Motor evoked potentials (MEP) recording timeline in all phases of the surgery.

Surgeon-directed MEP were monitored according to the local protocol. Transcranial electric stimulation was achieved and monitored using the Medtronic NIM-ECLIPSE (NIM-ECLIPSE® IONM system, Medtronic, MN, USA, Minneapolis). The MEP were evoked using needle electrodes placed in position C3/C4, and the stimulated cortex was below the anode. This positioning was according to the EEG standards, the international 10/20 system. A train of 5 pulses, with a duration of 0.20–1.00 ms, amplitude of max. 2×100 mA and an interstimulus interval of 2–3 ms with a frequency of 1 Hz were applied for stimulation. MEP were recorded with surface electrodes placed according to the standard approach muscle/tendon from both lower extremities (m. tibialis anterior and m. abductor hallucis). The correct placement of electrodes for MEP was checked in the prone position. The essential condition for MEP monitoring was recovery from neuromuscular blockade. First, baseline MEP parameters were recorded on the BIS level of 60 ± 5 and a train-of-four ratio of more than 90% before the commencement of surgery. The second MEP parameters were recorded when we deepened the sedation anesthesia to the BIS level of 40 ± 5 just before the skin incision. These two points were based on the recommended boundaries of a range of BIS index values to achieve an adequate depth of sedation in children and youth (29, 30). Other MEP were recorded after screws insertion, rods insertion, and spine correction (contraction, distraction). Surgeons´ interpretation was based on the local protocol. The protocol defines a significant change in MEP monitoring as MEP absence after stimulation. The absence of MEP is a warning criterion that informs about the possible risk of new neurological deficit development. We relativized all MEP parameters in percentage for the interindividual variability in MEP parameters among patients. The baseline amplitudes and latencies (BIS level 60 ± 5) were determined as 100%. Other amplitudes and latencies during the surgery were counted as percentages from the baselines. Figure 1 shows the MEP recording timeline. It is a graphical presentation of the electronic case report form (CRF).

### 2.5 Study objectives

The study’s primary objective was to evaluate the influence of the depth of sedation on the MEP reproducibility and interpretation by the surgical team in each patient separately. The secondary goal was to compare the relativized MEP parameters in percentage, latency, and amplitude at different levels of depth of sedation. These parameters were monitored in defined surgery phases. The initial MEP parameters were set as a baseline (100%), and other parameters were expressed as percentages. We evaluated MEP parameters and interpretations by surgeons for each patient separately. We did not compare the results of individual patients with those of other patients. So, the influence of different age groups on BIS varying values was excluded. We measured the incidence of new neurological deficits after surgery.

### 2.6 Statistical analysis

Standard descriptive statistics were applied in the analysis: absolute frequencies for categorical variables and mean, median, minimum, and maximum for continuous variables. We used the Statistica 13.5 software (STATISTICA software TIBCO Software, OK, USA, Tulsa). A non-parametric Wilcoxon Signed Ranks Test was performed to compare MEP parameters’ dependence on the BIS level 60 vs. 40.

## 3 Results

We started recruiting patients in September 2020. However, the recruitment was prolonged by a limited number of elective surgeries during the COVID-19 pandemic. The most frequent type of scoliosis was idiopathic scoliosis in 62% (*n* = 31) patients. Demographic and epidemiologic data are shown in Table 1. We achieved the appropriate depths of sedation in all children and youth before the skin incision.

**Table 1:** Patient’s characteristics, demographic, and epidemiologic data.

Patient’s characteristics	
	*n* = 50
Age, years, median (min–max)	15 (4–23)
Sex, F, *n* (%)	37 (74%)
Weight, kg, median (min–max)	50 (12–94)
Height, cm, median (min–max)	160 (97–188)
ASA^*^ physical status:	
ASA I, *n* (%)	20 (40%)
ASA II, *n* (%)	18 (36%)
ASA III, *n* (%)	12 (24%)
Scoliosis type:	
Idiopathic scoliosis, *n* (%)	31 (62%)
Neuromuscular scoliosis, *n* (%)	11 (22%)
Congenital scoliosis, *n* (%)	4 (8%)
Other scoliosis, *n* (%)	4 (8%)
Cobb’s angle, %, median, (min–max)	52 (0–109)
Operated segments, n, median, (min–max)	11 (3–18)

^*^ASA, American Society of Anesthesiologists.

Surgeons successfully recorded MEP in all patients during all phases of the surgery. The influence of a deeper level of sedation was not clinically significant, and MEP interpretation by a surgical team was not affected as a primary outcome.

The MEP parameters at BIS level 40 ± 5, proportionally compared with the baseline, were prolonged latencies 104% (97–110%), median (minimum – maximum), and decreased amplitudes 84.5% (51–109%). The absence of MEP was not observed at any level of sedation, BIS 40 ± 5 included. New neurological deficits did not develop in any of the patients. MEP parameters in all phases of surgery are illustrated in Table 2. We proved the statistically significant difference in latency and amplitude in dependence on the sedation depth. Results are presented in Table 3.

**Table 2:** Motor evoked potentials parameters (amplitude and latency) recorded in all phases of the surgery.

Motor evoked potentials	
	*n* = 50
Baseline (BIS* 60 ± 5)	
Reproducibility, *n* (%)	50 (100%)
Amplitude, %, median (min–max)	100 (100–100)
Latency, %, median (min–max)	100 (100–100)
Before incision (BIS 40 ± 5)	
Reproducibility, *n* (%)	50 (100%)
Amplitude, %, median (min–max)	84,5 (51–109)
Latency, %, median (min–max)	104 (97–110)
Screws (BIS (60–40) ± 5)	
Reproducibility, *n* (%)	50 (100%)
Amplitude, %, median (min–max)	94 (53–451)
Latency, %, median (min–max)	103 (85–109)
Rods (BIS (60–40) ± 5)	
Reproducibility, *n* (%)	50 (100%)
Amplitude, %, median (min–max)	96 (44–556)
Latency, %, median (min–max)	100 (78–108)
Dis/contraction (BIS (60–40) ± 5)	
Reproducibility, *n* (%)	50 (100%)
Amplitude, %, median (min–max)	96 (61–659)
Latency, %, median (min–max)	100 (81–110)

^*^BIS, Bispectral index.

**Table 3:** Statistically significant difference in motor evoked potentials parameters in dependence on the sedation depth.

	BIS* 60	BIS 40	BIS 40 × 60 difference	*p*-values
Amplitude (μV)	532.22 (345.37)	439.34 (290.45)	−92.88 (130.64)	< 0.001
Amplitude (%)	100 (0)	82.96 (14,28)	−17.04 (14.28)	< 0.001
Latency (ms)	31,06 (8,91)	32,24 (10,05)	1.18 (1.49)	< 0.001
Latency (%)	100 (0)	103.52 (3.49)	3.52 (3.49)	< 0.001

Mean (SD).

^*^BIS, Bispectral index.

## 4 Discussion

This prospective study describes the influence of sedation depth on MEP. To the best of our knowledge, this is one the largest and most significant sample of MEP parameters recorded in youth, so we decided to share these exciting data from the first 50 patients. Very few studies were completed on the influence of sedation depth on MEP. In the adult population, mostly during brain surgeries, the difference in sedation levels also resulted in MEP alteration. However, the number of studies and patient cohorts is very small, and further research should be performed to verify these results. Moreover, based on post-hoc power analysis, the data provided clinically significant results (31). In addition, our study is interesting because of the enrollment of all scoliosis types, not only the idiopathic, which is studied the most. The results apply to other types of scoliosis.

Although we described the statistically significant difference in MEP parameters in dependence on the sedation depth, the statistical alteration does not correlate with clinical alteration. These results imply that sedation depth in the recommended BIS range (40–60) ± 5 does not interfere with MEP interpretation by the surgical team, which is our most clinically significant finding. It supports the statement that surgeon-directed MEP are safe, feasible, and relatively simple method with high sensitivity and specificity (13, 16, 32). Although neurophysiologist-directed multimodal monitoring is the safest and preferred method, there is a limitation: a need for more skilled staff responsible for overseeing IONM. Many centers worldwide support the MEP monitoring certification not only for neurophysiologists but also for neuro-anesthesiologists or spinal surgeons. The advantage is that a skilled surgeon can record it alone without significant engagement during surgery (32–34). For these reasons, we consider this finding as the most clinically important.

Another clinically exciting result is that we could not prove the significant influence of the sedation depth in the recommended BIS range (60–40) ± 5 on the MEP amplitude and latency. The SCOL study’s strength is that it was designed to measure MEP in optimal conditions without significant influence of other variables, such as physiological or vital function status and surgical instrumentation. The only significant variables are the researched ones, e.g., defined doses of propofol and remifentanil, which affect the depth of sedation (17, 28, 35). The patients enrolled for the SCOL study were monitored as mentioned above. Appropriate ventilation, vasopressors, hemo/volume substitution, and body temperature management according to EBM were maintained within the normal values of vital function during the surgery (28).

As previous studies describe, most drugs used during anesthesia in children affect the IONM. Neuromuscular blocking agents should not be used during IONM except for the initial dose for intubation. Other unrecommended drugs are volatile anesthetics, benzodiazepines, or thiopental. These drugs in anesthetic doses significantly impair IONM (14, 36). A gold standard for maintaining general anesthesia during operations with IONM is considered a combination of propofol and opioids, which in clinically relevant doses reduce MEP amplitude and prolong the latency of SSEP, both insignificantly (19, 20). Although we have sufficient information about the influence of anesthetic agents on IONM, the exact effect of the depth of TIVA sedation on MEP is still unclear. BIS monitoring limitations result from using referential data from adult patients, and the suitability of EEG monitoring in pediatrics needs to be better described. However, when we set our research, we followed the most relevant studies at that time describing the influence of propofol on EEG changes in children. These changes were described as age dependent. However, the oscillations in the propofol-induced EEG were qualitatively similar among patients from 1 year old till adulthood, and BIS monitoring was commonly used off-label in children. In addition, some studies found no significant differences in BIS monitoring practice between pediatric and adult patients. In contrast, applying these devices to children younger than 1 year is not recommended (37–41). As scoliosis surgery is not indicated in children under 1 year, the most problematic group in BIS monitoring, we decided to use BIS in the same way as in adults. We decided to address the topic about the influence of depth of TIVA sedation on MEP in the SCOL study. As our findings show, the decrease of the amplitude was, on average, 15.5% (−9–49%), and the latency delay was, on average, 4.0% (−3–10%) at BIS level 40 ± 5. These results imply a minimal clinically significant influence on MEP amplitude and latency of sedation depth in the recommended range of BIS value 40–60 in youth. As BIS monitoring is considered a standard in surgeries under TIVA, and BIS in the range of 40–60 should be maintained, the effect of sedation probably should not be the leading cause of a sudden drop in amplitude or latency of MEP. On the other hand, as sedation levels of BIS under 40 are not recommended, these levels of sedation were not investigated. From other studies, we can assume that deeper sedation could significantly decrease amplitudes and prolonged latencies (31).

We could find a congruence for alarm points if we compare our results with studies by Benuska et al. and Kobayashi et al. The minor alarm points could be defined as a combination of an amplitude decrease of ≥70% and a latency delay of ≥10% from the baseline (42, 43). Although combined alterations of MEP parameters can reduce false positive values, these studies’ results are based on a small number of patients. A recent study by Magampa and Dunn and Polly et al. still recommend comparing amplitudes from legs and hands. It seems the loss of signal from the lower extremities and maintenance of signal from the upper extremities represent the most reliable method (13, 44). This claim is in consensus with the recommendation of the Scoliosis Research Society for IONM. The absence of signal both from the upper and lower extremities is likely due to technical issues or anesthetic implications. However, we did not measure this ratio because it was not our study outcome (9). If anesthetists maintain TIVA between the recommended range for sedation depth, the sudden drop in signal is more likely to be a technical issue and can be solved sooner to maintain patient safety (17).

A major study limitation was the number of enrolled patients. The COVID-19 pandemic limited elective surgery and recruitment of patients. The number of 50 patients from a planned sample size of 150 reduces the statistical analysis’s power. However, according to our research, this number represents a large sample of youths, and the presentation of the first 50 patients brings us interesting results, which could suggest ways to improve patient outcomes (42, 45). Another limitation is that we did not have target-controlled infusion (TCI) software for patients of all age and weight groups when we set this study protocol. Nevertheless, the EEG monitoring and maintenance of sedation between recommended levels is crucial for patient safety with both TCI and the calculated speed of continuous infusion. These are both possible ways to administer the propofol infusion (29). In addition, no significant differences were detected between continuous infusion guided by the BIS and TCI systems. We aimed to reduce the bias due to the choice of different models for different age groups (Kataria vs. Schnider). This study was designed when BIS monitoring was highly recommended during TIVA to prevent patients’ awareness during surgeries. These days, the reliability of the BIS value in predicting anesthesia that is too light is in question. Our study used a combination of BIS and clinical signs of patients responding to painful stimuli during surgery, such as elevation in heart rate, blood pressure, interference with artificial ventilation and patients´ movement to prevent the patients´ awareness. We had no suspicion of light sedation, and no intraoperative patient awareness was reported. Finally, although the EEG waveform in children varies with age, the BIS monitoring is not calibrated individually to age. However, based on previous studies in children and youth, we set these two points of the recommended range for BIS monitoring (29, 30). Regarding the reliability of BIS monitoring in children, the trend in sedation level could be described well with BIS. Also, the trend in sedation, thus in BIS, is important for the MEP trend. Our results did not show a significant drop in MEP, when the drop in BIS from 60 to 40 by deepening the sedation was performed. These results imply that drops in BIS in the recommended range should not clinically influence the MEP. The effect of the level of sedation which causes the burst suppression pattern on EEG was not part of this study, as this level of sedation is considered too deep and not recommended for general anesthesia. The more accurate monitoring of sedation levels in relation to MEP for example analysis of raw EEG or density spectral array analysis could be the theme for further research.

In conclusion, based on our preliminary data, it seems reasonable that surgeon-directed MEP may probably represent a promising way of neuromonitoring during scoliosis surgery in youth between the ages of 4 and 23 years. This method probably allows simple IONM alternatives when traditional neurophysiologist-led IONM is unavailable. However, our preliminary results should be interpreted cautiously because only a third of the patients were enrolled from the planned sample size, and we will continue with recruitment. The surgical team should always consider the pros and cons of the possibilities of IONM recording, especially in more complex cases with severe deformities, and choose the best option individually to maximize patient outcomes.

## Data Availability

The raw data supporting the conclusions of this article will be made available by the authors, without undue reservation.
